# Agricultural technology adoption and household welfare: Measurement and evidence

**DOI:** 10.1016/j.foodpol.2019.101742

**Published:** 2019-08

**Authors:** Tesfamicheal Wossen, Arega Alene, Tahirou Abdoulaye, Shiferaw Feleke, Victor Manyong

**Affiliations:** a International Institute of Tropical Agriculture (IITA), Nairobi, Kenya; b IITA, Lilongwe, Malawi; c IITA, Ibadan, Nigeria; d IITA, Dar es Salaam, Tanzania

**Keywords:** Adoption, Bias, DNA, Misclassification, Nigeria, Welfare

## Abstract

Previous studies on the adoption and impacts of improved crop varieties have relied on self-reported adoption status of the surveyed households. However, in the presence of weak variety maintenance and poorly functioning seed certification system, measurement errors in self-reported adoption status can be considerable. This paper investigates how such measurement errors can lead to biased welfare estimates. Using DNA-fingerprinting based varietal identification as a benchmark, we find that misclassification in self-reported adoption status is considerable, with significant false negative and positive response rates. We empirically show that such measurement errors lead to welfare estimates that are biased towards zero and substantially understate the poverty reduction effects of adoption. While the empirical evidence suggests attenuation bias, our theoretical exposition and simulations demonstrate that upward bias and sign reversal effects are also possible. The results point to the need for improved monitoring of the diffusion process of improved varieties through innovative adoption data collection approaches to generate robust evidence for prioritizing and justifying investments in agricultural research and extension.

## Highlights

• Self-reported and DNA-fingerprinted adoption status compared.
• Substantial misclassification in self-reported adoption status.
• Misclassification leads to biased welfare estimates.
• Improved monitoring of the diffusion process of improved varieties is crucial.

## 1 Introduction

What is the welfare effect of adopting high-yielding crop varieties (HYVs)? This question has generated a lot of interest in the development economics literature. Consequently, numerous studies have assessed the impacts of HYVs on welfare-related outcome indicators (Evenson and Gollin, 2003, Thirtle et al., 2003, Minten and Barrett, 2008, Rusike et al., 2010, Dercon and Christiaensen, 2011, Suri, 2011, Shiferaw et al., 2014, Zeng et al., 2015, Verkaart et al., 2017). The empirical evidence on the link between technology adoption and welfare-related indicators suggests that there are potentially large gains from adoption through direct and indirect pathways (Thirtle et al., 2003, Alene and Coulibaly, 2009, Christiaensen et al., 2011, Pingali, 2012, Bezu et al., 2014, Smale and Mason, 2014, Shiferaw et al., 2014, Mathenge et al., 2014, Zeng et al., 2015, Khonje et al., 2015, Alwang et al., 2019). These include direct benefits through productivity gains and indirect benefits through output, input and labor market adjustments. Adopters can experience income gains directly if productivity gains are larger than subsequent price falls. Even when prices are falling, large productivity gains are still critical because most adopters are both producers and consumers due to prevalent market imperfections^1^. Indirect benefits include productivity growth-induced lower food prices, particularly for net-food buyers, and employment opportunities for the poor and landless farmers (Zeng et al., 2015, Kassie et al., 2017, Alwang et al., 2019).

However, the empirical evidence so far has relied on self-reported adoption data by directly eliciting information on improved variety names and adoption status from farmers. In the presence of weak and poorly-regulated extension and seed systems, measurement errors in self-reported adoption status can be considerable. In fact, using a novel DNA-fingerprinting based varietal identification approach, numerous studies (Wossen et al., 2019, Kosmowski et al., 2019, Maredia et al., 2016, Ilukor et al., 2017, Wossen et al., 2019) have documented the presence of significant measurement errors in self-reported adoption data. However, there is little evidence on whether and how misreporting adoption status generates spurious findings. This has significant implications for drawing robust policy recommendations, since policy makers typically use such results to alter, improve or even design new programs that have far-reaching consequences on the livelihoods of farmers.

This paper examines whether and how misreporting adoption status can lead to biased welfare estimates. Unlike welfare indicators, such as consumption data, which is typically a continuous variable, adoption status is often measured by a binary variable. However, measurement error in a binary variable is always non-classical. In our context, measurement error in adoption status may lead to biased estimates in two ways. The first is the non-classical nature of the measurement error. Second, the measurement error itself can also be endogenous as households that misreport adoption status can be different from those who correctly report their adoption status in both observed and unobserved characteristics (Wossen et al., 2019). Therefore, estimating the causal effect of adoption on welfare outcomes is not straightforward, even when the usual exclusion restrictions are met. In this regard, Nguimkeu et al. (2019) and Wossen et al. (2019) show that endogenous misclassification can lead to upward or downward biases as well as sign reversal effects.

To our knowledge, no parametric identification strategy currently exists for a misreported endogenous treatment variable when misreporting is bidirectional and possibly endogenous. While previous studies have relied non-parametric approaches (Lewbel, 2007, Mahajan, 2006, Hu and Schennach, 2008), this paper aims to overcome measurement error related biases using validation data. While the existing literature uses administrative data as a benchmark, which continues to be prone to measurement errors, our study uses a novel data collection approach that minimizes measurement error in the benchmark data. This is achieved by collecting improved variety adoption data from households at a specific point in time in two different ways. First, we collected information by asking households to report the type of variety they grow, specifically whether the crop variety they grow is improved or not. This corresponds to the standard data collection approach often employed in household surveys. We then took leaf samples from each respondent’s plot to identify the type of varieties they grow through DNA-fingerprinting analysis. Since the DNA-fingerprinting approach is independent of environmental conditions or plant growth stage, the type of the variety grown by an individual farmer can be identified accurately (Rabbi et al., 2015). Therefore, the DNA-fingerprinted adoption data serves as an independent validation data (Bollinger and David, 1997). Since we have this validation data alongside self-reported data, we consider adoption status based on DNA-fingerprinting as a benchmark. The bias caused by endogenous misreporting of adoption status is, therefore, determined by the difference in the estimates of the benchmark and the error-ridden self-reported adoption data.

This paper relates and contributes to the literature on measurement errors in general (Lewbel, 2007, Mahajan, 2006, Nguimkeu et al., 2019) and on how non-classical measurement errors in household surveys may affect statistical inference, in particular (Carletto et al., 2013, Gourlay et al., 2017, Desiere and Jolliffe, 2018, Beegle et al., 2012, Abay et al., 2019). In addition, this paper also provides new insights into the literature on technology adoption and welfare (Shiferaw et al., 2014, Zeng et al., 2015, Verkaart et al., 2017). The key findings of this paper are as follows: First, misclassification of adoption status is considerable, with significant false negative and positive rates. Second, such measurement errors in self-reported adoption status generate welfare estimates that are biased towards zero and substantially understate the poverty reduction effects of adoption. Third, while our empirical evidence shows attenuation bias, our theoretical exposition and simulations suggest that upward bias and sign reversal effects are also possible.

The rest of the paper is structured as follows. Section 2 presents the theoretical framework on measurement error. Section 3 presents the data source, the descriptive statistics and the empirical strategy. Section 4 presents the main results and Section 5 concludes with implications for future research.

## 2 Context on measurement error

This section presents the theoretical framework by explicitly linking technology adoption with welfare outcomes. The framework extends the approach of Wossen et al. (2019) by considering non-classical measurement error in both the outcome and treatment variables. Let $W_{i}^{*}$ be the true level of welfare (consumption expenditure in our case) enjoyed by a given household and $T_{i}^{*}$ be the true adoption status as measured by DNA-fingerprinting. Assuming a linear relationship, the true welfare level of the household is expressed as a function of true adoption status in the following manner^2^.

(1) $$W_{i}^{*}=\delta T_{i}^{*}+\mu_{i}$$

In the above specification, our parameter of interest δ cannot be identified due to the endogeneity of the adoption variable (i.e., we are assuming that $\mathrm{cov}(T_{i}^{*},\mu_{i})\;\neq \;0$). We therefore explicitly model the true adoption decision of households as follows:

(2) $$T_{i}^{*}=1[\varphi \prime z_{i}+\omega_{i}>0]$$

where $z_{i}$ is a vector of exogenous determinants of adoption including the identifying instruments, such that $E[\mu_{i}|z_{i}]=0$.

In the above specification, while $T_{i}^{*}$ is endogenous, it is not misclassified. Therefore, δ can be identified using an IV regression approach. However, in the absence of a gold standard benchmark such as DNA-fingerprinting, the researcher only observes $T_{i}$, the farmer’s self-reported adoption status which is potentially a misclassified version of the true treatment status, $T_{i}^{*}$. The reported adoption status, $T_{i}$, can be measured with error because some true adopters may report non-adopter status (false negatives) and some true non-adopters may report adopter status (false positives). The relationship between $T_{i}^{*}$ and $T_{i}$ can be specified as follows:

(3) $$T_{i}=T_{i}^{*}+\eta_{i}$$

(4) $$\eta_{i}=\left\{\begin{array}{cc} -1, & \mathrm{falsenegatives}\text{.}\\ 0, & \mathrm{correctclassification}\text{.}\\ 1, & \mathrm{falsepositives}\text{.} \end{array}\right)$$

As can be seen from Eq. (4), the measurement error, $\eta_{i}$, takes on values (−1, 1) in the presence of misclassification and a value of zero, otherwise. This measurement error is non-classical as it is necessarily negatively correlated with the underlying true treatment status (i.e., $\mathrm{cov}(T_{i}^{*},\eta_{i})<0$). Moreover, the measurement error, $\eta_{i}$, is likely to be endogenous (i.e. $\mathrm{cov}(\eta_{i},\mu_{i})\;\neq \;0$), so that the self-reported adoption status, $T_{i}$, is also endogenous (i.e., $\mathrm{cov}(T_{i},\mu_{i})\;\neq \;0$). Using Eq. (1) and Eq. (3), the OLS estimator of the treatment effect δ^ols is given by:

(5) δ^ols=cov(Ti,Wi∗)var(Ti)=δ[σT∗η+σT∗2]+σT∗μ+σημσT∗2+2σT∗η+ση2

In our case, both adoption status and misclassification are likely to be endogenous and hence the sign of the bias in δ^ols is unknown. Assuming exogenous adoption decision and misreporting (i.e., $\sigma_{T^{*}\mu }=0,\;\sigma_{\eta \mu }=0$), then δ^ols will be biased towards zero. For example, if better-off households report non-adopter status when they are indeed adopters, then OLS estimates will be biased towards zero. On the contrary, if better-off households report adopter status when they are truly non-adopters, then OLS estimates will be biased upwards.

Furthermore, δ will still not be identified, even when a valid instrument is available. Specifically, suppose that $z_{i}$ is a valid and exogenous instrument for $T_{i}^{*}$, as defined earlier. The IV estimator for self-reported adoption data (δ^iv) is obtained using two-stage least squares in the following manner. In the first-stage, the predicted value of $T_{i}$ (i.e. $E[T_{i}|z_{i}]$) is estimated. In the second-stage, $T_{i}$ is replaced by $E[T_{i}|z_{i}]$. Hence, the bias in δ^iv is given by:

(6) δ^iv=cov(Wi∗,E(Ti|zi))var(E(Ti|zi))=cov[E(Ti|zi),E(Ti∗|zi)]var(E[Ti|zi])=δcovE[Ti|zi],Fω(ziφ)var(E[Ti|zi])

where $F_{\omega }(.)$ is the cdf of ω, and $F_{\omega }(z_{i}\varphi )$ is the conditional expectation of $T_{i}^{*}$ implied by Eq. (2). Eq. (6) shows that, δ^IV is biased in unknown ways.

While our main focus is on the measurement of adoption status, measurement error in consumption expenditure data (outcome variables) may also be pervasive. If the measurement error in the expenditure data is non-classical, then δ^ols will be biased. For example, if farmers who are better at identifying improved varieties are also likely to be better at reporting consumption data, then the measurement error in adoption and welfare variables will be correlated. Suppose that instead of the true household welfare($W_{i}^{*}$) only a misreported measure ($W_{i}$) is observed by the researcher such that $W_{i}=W_{i}^{*}+\Psi_{i}$. Suppose further, that the measurement error $\Psi_{i}$ is non-classical such that we can write $\Psi_{i}=\alpha W_{i}^{*}+\nu_{i}$. In the presence of non-classical measurement error in Wi∗,δ^ols becomes:

(7) δ^ols=cov(Wi,Ti∗)var(Ti∗)=δ(1+α)

The above equation suggests that if the adoption decision is strictly exogenous and the measurement error in consumption expenditure is not correlated with adoption status, then δ^ols=δ(1+α) and the proportional bias is α. 3 Since in our context, we expect that δ⩾0, the sign of the bias due to possible non-classical measurement error in consumption expenditure would be the sign of α, which would typically be negative, given the mean-reverting nature of the measurement errors in consumption and earnings data as consistently found in the literature (Bound and Krueger, 1991, Bollinger, 1998, Gibson et al., 2017). If the instruments, $z_{i}$, for true adoption and $T_{i}^{*}$ are exogenous to both $\mu_{i}$ and $\nu_{i}$, then following the same reasoning as above, the IV estimator using the self-reported data is δ^iv=δ(1+α)λ, where λ is the covariance term in Eq. (6). Similarly, the IV estimator using the true adoption data is δ^iv=δ(1+α), and its proportional bias is α. 4

## 3 Data and empirical strategy

### 3.1 Data

This study is conducted in Nigeria. We focus on the adoption of improved cassava varieties because Nigeria is the largest cassava producer in the world. Cassava is the most widely cultivated root crop in terms of area allocation and the number of growers in the country, which justifies our focus on the crop. To collect a nationally representative data, a multistage stratified sampling design is used. First, the list of Enumeration Areas (EAs) are obtained from the National Population Commission (NPC). From each region, 125 EAs are selected using probability proportional to size (PPS) sampling approach. From each EA, five cassava growing households are randomly selected, resulting in a sample size of 2500 households. During the post-planting survey, we asked each household to specify the name of the cassava variety they grow and whether the variety is local or improved. We collected this information at the variety and plot level. Following the collection of self-reported adoption data, we visited all cassava plots of the respective farmer and collected leaf samples for DNA-fingerprinting analysis.^5^

We then matched self-reported adoption status from the household survey with the DNA-fingerprinted adoption data to determine the rate of misclassification (false positives and negatives). Using the household survey data, we find that about 59% of the households have adopted improved cassava varieties.^6^ However, when adoption is measured using DNA-fingerprinting approach, the adoption rate is 66%. Using DNA-fingerprinted adoption data as a benchmark, we find 15% and 19% false positive and negative responses, respectively.^7^ These results suggest that both false positive and negative responses are prevalent. This result is consistent with other studies that documented significant misidentification of varieties by farmers (Kosmowski et al., 2019, Maredia et al., 2016, Ilukor et al., 2017).

While examining the correlates of misreporting is not the objective of this paper, our exploratory analysis suggests that the current weak and dysfunctional variety release procedures and cassava seed system might have played a role for the observed high variety misidentification rates by farmers (Wossen et al., 2019). In Nigeria, the cassava seed system remains largely informal. According to our data, for about 70% of the farmers, the primary source of planting material is the informal system (own- saved stems, exchange with friends, relatives, and neighbors). Further, a lack of proper and consistent variety naming is an issue. For example, in our data we find that most farmers give the same name for different varieties and different names for the same variety. This paper, therefore, examines how such misclassfication (false negative and positive responses) may bias parameter estimates of welfare related outcome indicators.^8^

### 3.2 Definition of key variables

#### 3.2.1 Adoption variable

As mentioned above, our main treatment variable, adoption of improved varieties, is measured in two ways. The first, which is the benchmark, is using DNA-fingerprinted data and the second is using self-reported data. Since our welfare indicators are measured at the household level, we also aggregated adoption measures at the household level. For the benchmark data, the treatment variable takes a value of one if the farmer grows at least one improved variety in one of his/her plots, as confirmed by the DNA-fingerprinting analysis and zero otherwise. Similarly, for the household survey, the treatment variable takes a value of one if the farmer reports adoption status in at least one of his/her plots and zero otherwise. As a robustness check, we also measured the above treatment variables at the plot level. In this case, the treatment variable assumes a value of one if the plot is under an improved variety and zero otherwise^9^.

#### 3.2.2 Welfare variables

In this paper, we use food availability and consumption expenditure (food and total consumption expenditure) as a measure of welfare. Unlike adoption data, our welfare indicators come from recall data.^10^ Our survey included an extensive consumption module. In this module, we collected data on food shortages and expenditure on food and non-food items. The food shortage indicator is constructed using the following question: *“Were there any month(s), in the past 12 months, in which you did not have enough food to meet your family’s needs?”* This includes any kind of food from any source, such as own production, purchase or exchange, food aid, or borrowing. Based on the above question, we created a dummy variable that takes on a value of one if the household did not face any food shortage in any of the months in the past 12 months and zero otherwise. This indicator, we believe, captures at least three of the four pillars of food security.^11^ Since it is framed to include own production and purchased food item from the market, it captures the availability and accessibility dimensions of food security. In addition, since the question considers food availability throughout the whole season, it also captures the temporal aspects of food security. Our survey suggests that about 63% of the respondents faced food shortages during the survey season. This rate is higher among non-adopters (79%) compared to adopters (52%).

**Fig. 1:**
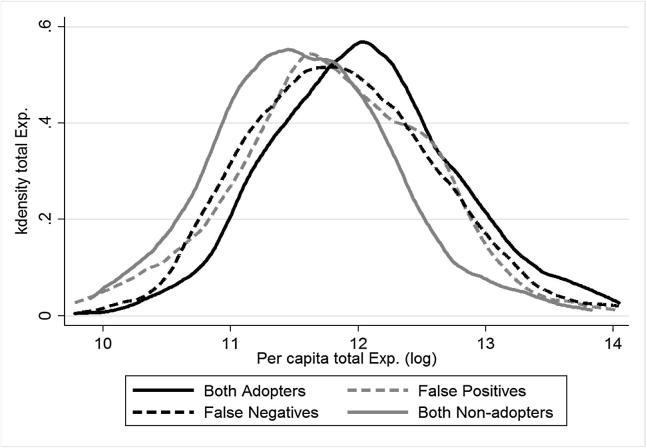
Total expenditure by misclassification status.

Further, we collected food expenditure data based on seven day recalls for more than 164 food items. Food expenditure is comprised of monetary expenditures on purchased food, as well as food consumed from own production and received from other sources such as gifts and food aid. We calculated the monetary value of food consumption from own production and gifts using household-specific unit values. Unit values are observed for households that purchased the food items in the past seven days. When household-specific prices were not available, we used community-level median price. For non-food expenditure, we collected data from more than 42 items. Total consumption expenditure was then calculated as the sum of food and non-food expenditure. Fig. 1 shows the distribution of per-capita total expenditure by self-reported and DNA-fingerprinted adoption status. The distribution has some interesting features. First, adopters have higher per-capita expenditure than non-adopters. Second, given that the distribution is shifted to the right, false negatives have higher per-capita expenditure compared to non-adopters. Third, the per-capita expenditure of false positives is lower than that of actual adopters and false negatives. These suggest that misclassification and welfare outcomes are correlated.

#### 3.2.3 Other socio-economic characteristics

Table 1 presents the descriptive statistics of other control variables. Household characteristics such as age, household size and education, as well as social capital in terms of membership in different social groups, alongside a number of wealth indicators including livestock ownership expressed in terms of tropical livestock units (TLU) are included to control for possible heterogeneity between adopters and non-adopters.

**Table 1:** Socio-economic characteristics of the sample households by DNA-fingerprinting adoption status.

	All	Adopters	Non-adopters	Mean diff
Household size (#)	4.51	4.63	4.30	0.33^∗∗∗^
Number of boys below 12	1.22	1.22	1.23	−0.008
Number of girls below 12	1.33	1.29	1.41	−0.11^∗^
Education (Years)	8.86	9.15	8.36	0.80^∗∗∗^
Age (Years)	51.20	51.79	50	1.79^∗∗∗^
Sex (1 = Male)	0.89	0.88	0.91	−0.04^∗∗∗^
Livestock ownership (TLU)	0.61	0.80	0.26	0.54
Total land size (ha)	6.54	4.90	5.37	−0.47
Value of asset ($ US)	937	993	840	153
Television ownership (1 = yes)	0.73	0.75	0.68	0.07^∗∗∗^
Mobile phone ownership (1 = yes)	0.96	0.97	0.94	0.04^∗∗∗^
Access to off-farm (1 = yes)	0.26	0.28	0.23	0.05
Access to extension (1 = yes)	0.36	0.39	0.32	0.07^∗∗∗^
Access to credit (1 = yes)	0.44	0.46	0.41	0.05^∗∗^
Member to cassava association (1 = yes)	0.21	0.22	0.18	0.04^∗∗^
Informal credit and saving (1 = yes)	0.33	0.34	0.30	0.04^∗∗^
Membership to cooperatives (1 = yes)	0.25	0.27	0.22	0.05^∗∗^
Incidence of cassava pests (1 = yes)	0.25	0.30	0.18	0.12^∗∗∗^
Garri and fufu preference (1 = most important traits)	0.59	0.7	0.41	0.29^∗∗∗^
Road quality^a^	3.28	3.35	3.16	0.19^∗∗^
Distance from village market (km)	3.07	2.87	3.40	−0.53^∗∗∗^
Distance from district market (km)	12.64	11.59	14.47	−2.88^∗∗∗^
Distance from fertilizer dealer (km)	12.00	11.31	13.20	−1.88^∗∗∗^
N	2214	1401	811	

Significance codes: ^∗∗∗^1%, ^∗∗^5%, and ^∗^10%.

a It ranges from very poor(1) to very good (5).

### 3.3 Empirical strategy

Our empirical strategy builds on the theoretical framework presented in Section 2 and estimates the relationship between adoption and household welfare indicators in the following manner:

(8) $$W_{i}=\alpha_{0}+\gamma T_{i}+\beta^{\prime }X_{i}+\vartheta^{\prime }V_{J}+\varepsilon_{i}$$

where $W_{i}$ is an outcome indicator measuring welfare (consumption expenditure and food shortage indicators in this context). $T_{i}$ takes a value of one if the farmer reports adoption status and zero otherwise. *X* includes a vector of household characteristics reported in Table 1. *V* captures other controls at the village level (location dummies). In the above welfare function, γ measures the welfare effect of adoption. The above empirical specification is then re-estimated using DNA-fingerprinted adoption status ($T_{i}^{*}$) instead of ($T_{i}$).

(9) $$W_{i}=\alpha_{0}+\gamma T_{i}^{*}+\beta^{\prime }X_{i}+\vartheta^{\prime }V_{J}+{∊}_{i}$$

We estimate the above models using simple OLS. The size and direction of γ^, the OLS estimator of the model with self-reported adoption status, and γ^∗, the OLS estimator of the model with DNA-fingerprinted data measures the bias caused by exogenous misreporting (assuming that adoption decision is also exogenous). If γ^∗>γ^, misclassification leads to attenuation bias. If γ^∗<γ^, we expect an upward bias. If γ^∗ and γ^ assume opposite signs, then misclassification leads to sign reversal effects.

However, the above specifications do not take into account the endogeneity of the adoption decision. The adoption decision can be endogenous as adopters might be significantly different from non-adopters in key unobservable characteristics such as management ability, farming and varietal identification skills which are likely to be correlated with the adoption decision and welfare outcome indicators. As such, identifying the causal effect of adoption on welfare outcomes requires exogenous sources of variation for the adoption variable. For our identification strategy, we use consumption trait preference heterogeneity and village-level incidence of cassava pests as instruments. In particular, we used consumption preference heterogeneity related to *fufu* and *gari* by eliciting information on consumption traits farmers identify as important. *Fufu* and *gari* are the two most important uses of cassava in Nigeria. In general, new and improved varieties tend to have not only better yield and diseases resistance capacity but also better *fufu* and *gari* quality. Without adoption of improved cassava varieties, preference heterogeneity in *fufu* and *gari* traits cannot affect welfare outcomes since these are traits that are peculiar to cassava. Our second instrument, the incidence of cassava pests, is measured at the village level with the presumption that village level shock would be exogenous to individual household characteristics. Our first stage regression result suggests that the instruments are relevant as they are significant at 1% level.

Finally, since our empirical approach doesn’t take into account the endogeneity of the measurement error, we introduce $\eta_{i}$ as an additional control in the above regression specifications. By controlling for $\eta_{i}$, we can overcome the possible endogeneity of the measurement error, even when the sources of the measurement error are unknown. As such, we estimate the following regression specification:

(10) $$W_{i}=\alpha_{0}+\gamma T_{i}+\beta^{\prime }X_{i}+\vartheta^{\prime }V_{J}+\theta \eta_{i}+\varepsilon_{i}$$

## 4 Results

In this section, we present our main results in three sub-sections. The first section presents OLS estimates, while the second and third sections present IV estimates and robustness checks, respectively.

### 4.1 OLS estimation results

Table 2 presents OLS estimates based on self-reported and DNA-fingerprinted adoption data. Herein, we first focus on OLS results assuming an exogenous adoption decision and misreporting. In both Panel A and B, we address the endogeneity of the measurement error by controlling for $\eta_{i}$. In this case, parameter estimates will be unbiased, even when the sources of the measurement error are unknown as long as the adoption decision is strictly exogenous. Results reported in Table 2 show that adoption has a positive and statistically significant effect on welfare indicators in both the self-reported and DNA-fingerprinted adoption data.^12^ Even though the statistical significance is the same, the economic significance is quite different. In particular, our results suggest significant attenuation bias due to misclassification. For example, we find that the probability of experiencing food shortage declines by 26.2% when using self-reported adoption data compared to about 34% when using the DNA-fingerprinted adoption data. More importantly, the pattern of the attenuation bias is consistently similar across all consumption expenditure indicators. These results are consistent with the findings of Aigner, 1973, Lewbel, 2007. As expected, in the regression specifications where we controlled for misreporting (i.e., $\eta_{i}$), estimated welfare effects are the same when using self-reported and DNA-fingerprinted adoption data (i.e., δ^OLS($T_{i}$) - δ^OLS($T_{i}^{*}$) = 0).

**Table 2:** OLS estimates.

	Food shortage	Food exp.	Total exp.	Food shortage	Food exp.	Total exp.
	Self-reported (Panel A)					
Adoption ($T_{i}$)	−0.262^∗∗∗^	0.191^∗∗∗^	0.142^∗∗∗^	−0.512^∗∗∗^	0.460^∗∗∗^	0.360^∗∗∗^
	(0.026)	(0.041)	(0.035)	(0.026)	(0.048)	(0.041)
Measurement error ($\eta_{i}$)				0.309^∗∗∗^	−0.334^∗∗∗^	−0.271^∗∗∗^
				(0.023)	(0.034)	(0.029)
						
R^2^		0.295	0.320		0.320	0.342
Pseudo R^2^	0.106			0.116		
						
	DNA-fingerprinted (Panel B)					
Adoption ($T_{i}^{*}$)	−0.340^∗∗∗^	0.353^∗∗∗^	0.284^∗∗∗^	−0.512^∗∗∗^	0.460^∗∗∗^	0.360^∗∗∗^
	(0.022)	(0.034)	(0.029)	(0.026)	(0.048)	(0.041)
Measurement error ($\eta_{i}$)				−0.203^∗∗∗^	0.126^∗∗∗^	0.090^∗∗∗^
				(0.026)	(0.040)	(0.034)
						
R^2^		0.320	0.342		0.323	0.344
Pseudo R^2^	0.154			0.134		
						
δ^OLS($T_{i}$) - δ^OLS($T_{i}^{*}$)	0.076^∗∗^	−0.163^∗∗∗^	−0.142^∗∗∗^	0.00	0.00	0.00
	(0.034)	(0.048)	(0.04)			
						
Others controls	Yes	Yes	Yes	Yes	Yes	Yes
Location dummies	Yes	Yes	Yes	Yes	Yes	Yes
N	2,214	2,214	2,214	2,214	2,214	2,214

Standard errors clustered at the enumeration area-level are reported in parentheses.

Other controls include the variables listed in Table 1.

Significance codes: ^∗∗∗^ 1%, ^∗∗^ 5%, and ^∗^ 10%.

Coefficients for food shortage are treatment effects from bivariate probit model.

However, in our case, the adoption decision is unlikely to be exogenous. Therefore, we report IV results in the next section. Note that, our IV regression addresses the endogeneity of the adoption decision but not the measurement error.^13^ Hence, we also included $\eta_{i}$ as an additional control in our IV regressions.

### 4.2 IV estimation results

Our IV regression results are presented in Table 3.^14^ In this specification, we instrumented for the endogeneity of the adoption variable. Therefore, our approach can only address the endogeneity of the adoption decision, not the endogeneity of the measurement error. The endogeneity of the measurement error will only bias estimates of the self-reported adoption data. Since the DNA-fingerprinted adoption data is assumed to be free from measurement error, IV estimates will be unbiased. However, the IV estimates from the household survey will still be biased due to the endogeneity of the measurement error. The IV result from the DNA-fingerprinted data is therefore our benchmark. We draw some very interesting insights based on results reported in Table 3: Endogenous misclassification leads to attenuation bias as the effect size of the adoption variable is smaller when using self-reported adoption data compared to DNA-fingerprinted adoption data for all welfare outcome indicators. For example, for food shortage indicator, the effect is attenuated by 12.4 percentage point due to misclassification. Estimates for consumption expenditure indicators are also attenuated due to misclassification. Ignoring misclassification would thus provide a highly misleading conclusion on the economic significance of key policy variables.^15^

**Table 3:** IV estimates.

	Food shortage	Food exp.	Total exp.	Food shortage	Food exp.	Total exp.
	Self-reported (Panel A)					
Adoption ($T_{i}$)	−0.240^∗∗∗^	0.41^∗∗∗^	0.287^∗∗^	-0.320^∗∗^	0.593^∗∗∗^	0.427^∗∗∗^
	(0.084)	(0.148)	(0.122)	(0.144)	(0.179)	(0.149)
Measurement error ($\eta_{i}$)				0.082^∗∗∗^	-0.391^∗∗∗^	-0.299^∗∗∗^
				(0.034)	(0.079)	(0.067)
						
R^2^		0.295	0.320		0.321	0.343
						
	DNA-fingerprinted (Panel B)					
Adoption ($T_{i}^{*}$)	−0.364^∗∗^	0.592^∗∗^	0.414^∗∗^	-0.362^∗∗∗^	0.585^∗∗^	0.449^∗∗^
	(0.104)	(0.265)	(0.219)	(0.118)	(0.256)	(0.212)
Measurement error ($\eta_{i}$)				-0.103	0.197	0.140
				(0.26)	(0.154)	(0.126)
						
R^2^		0.320	0.342		0.321	0.344
						
δ^IV($T_{i}$) - δ^IV($T_{i}^{*}$)	0.124^∗∗^	-0.182^∗∗∗^	-0.127^∗∗∗^	0.042	0.008	-0.022
	(0.035)	(0.05)	(0.042)	(0.06)	(0.01)	(0.08)
						
Others controls	Yes	Yes	Yes	Yes	Yes	Yes
Location dummies	Yes	Yes	Yes	Yes	Yes	Yes
N	2,214	2,214	2,214	2,214	2,214	2,214

Standard errors clustered at the enumeration area-level are reported in parentheses.

Other controls include the variables listed in Table 1.

Significance codes: ^∗∗∗^ 1%, ^∗∗^ 5%, and ^∗^ 10%.

Even though our results suggest the presence of an attenuation bias, measurement error in self-reported adoption data can also lead to an upward bias or sign reversal effects. In our case, the attenuation bias can be explained by the higher proportion of false negatives relative to false positives. In particular, the higher estimated welfare outcomes when using DNA-fingerprinted adoption data are likely to be due to the improved genetics effect (as false negatives have an improved variety), which appears to be stronger than gains through behavioral adjustments by false positive groups (Note that, in the DNA-fingerprinted sample, the adopter group includes correct improved variety identifiers and false negatives. On the other hand, the adopter group in the self-reported sample includes correct improved variety identifiers and false positives). In all of the regression specifications, where we included the measurement error, $\eta_{i}$, as an additional regressor, the estimated welfare effects are the same when using self-reported and DNA-fingerprinted adoption data (i.e., δ^IV($T_{i}$) - δ^IV($T_{i}^{*}$) = 0). Furthermore, the estimated coefficient on $\eta_{i}$ is consistently insignificant for the DNA-fingerprinting sample, which suggests that the DNA-fingerprinting is a more accurate measure of the true adoption status of farmers in our sample.

What is the implication of misreporting in estimating the poverty reduction effects of adoption? We establish this link using the international poverty line of $1.9 PPP per capita per day(World Bank, 2011, Ravallion et al., 2009). Results are reported in Table 4.^16^

**Table 4:** Misclassification and poverty.

	Poor (1 = Yes)	Poor (1 = Yes)
Adoption (Self-reported)	−0.238^∗∗∗^	
	(0.083)	
Adoption (DNA)		−0.364^∗∗∗^
		(0.104)
		
Others controls	Yes	Yes
Location dummies	Yes	Yes
N	2214	2214

Bootstrapped standard errors are reported in parentheses.

Other controls include the variables listed in Table 1.

Significance codes: ^∗∗∗^ 1%, ^∗∗^ 5%, and ^∗^ 10%.

The result indicates that adoption reduces the probability of being poor but the economic significance of the effect differs drastically depending on the way adoption is measured. While the estimate from the self-reported adoption data suggests a 23.8% reduction in the probability of being poor, the estimate from the DNA-fingerprinted adoption data suggests a 36.4% reduction in the probability of being poor. This implies that estimates on the poverty reduction effects of adoption are sensitive to the measurement of adoption status and hence improved monitoring of the diffusion process of improved varieties through innovative adoption data collection approaches is crucial for prioritizing and justifying investments in the agricultural research and extension (Wossen et al., 2019).

### 4.3 Robustness check

To probe the robustnes of our main results, we conduct the following three robustness checks: First, we relax the exogeneity assumption in Section 4.2 using an alternative identification strategy proposed by Lewbel (2012). The Lewbel (2012) approach exploits model heteroscedasticity to construct instruments using other regressors in the model. Second, we demonstrate the magnitude and sign of misclassification bias under different shares of endogenous bidirectional misclassification through Monte Carlo simulations. Third, to check the consistency of our main household level results, we also present estimates at the plot level.

#### 4.3.1 Alternative identification strategy

In this section, we present alternative estimates using the Lewbel (2012) approach. Results are presented for per-capita total expenditure using self-reported and DNA-fingerprinted adoption data. Results reported in Table 5 suggest that the size of the estimated coefficients from Lewbel (2012) approach are very similar with those reported in Table 3.

**Table 5:** Results from an alternative identification strategy.

	Self-reported	DNA-fingerprinted
Adoption	0.351^∗∗∗^	
	(0.114)	
Adoption		0.42^∗∗∗^
		(0.09)
		
Others controls	Yes	Yes
Location dummies	Yes	Yes
N	2214	2214

Bootstrapped standard errors are reported in parentheses.

Other controls include the variables listed in Table 1.

Significance codes: ^∗∗∗^ 1%, ^∗∗^ 5%, and ^∗^ 10%.

#### 4.3.2 Monte Carlo simulations

In this section, we demonstrate the size and sign of misclassification bias through Monte Carlo simulations. In our simulation setup, we use the data generation process described by Eqs. (1), (2), (3) in Section 2 for welfare, true adoption and observed adoption status, respectively. The error terms $\mu_{i},\omega_{i}$ and $\eta_{i}$ are drawn from a trivariate normal distribution. The correlations between $\left(\mu_{i},\omega_{i}),\;(\mu_{i},\eta_{i}\right)$ and $\left(\eta_{i},\omega_{i}\right)$ are denoted by $\sigma_{\mu \omega },\;\sigma_{\mu \eta }$ and $\sigma_{\omega \eta }$, respectively. In the simulation, we vary the proportion of false negatives ∈{0,0.3,0.47,0.5}, false positives $\in \{0,0.05,0.1,0.25\},\sigma_{\mu \omega }\in \{-0.8,0.1,0.01,0,0.8\},\sigma_{\mu \eta }\in \{-0.3,0.01,0,0.3\}$ and $\sigma_{\omega \eta }\in \{-0.3,0,0.3\}$. Finally, we set the true welfare effect of adoption at 30%.

As shown in Table 6, the OLS estimator produces estimates that are biased towards zero or upwards. In some cases, the OLS estimator produce estimates with the opposite sign compared to the true treatment effect. At high level of misclassification and endogneity (with a negative $\sigma_{\mu \omega }$ and $\sigma_{\mu \eta }$), the attenuation bias is so strong that the sign of the treatment effect is reversed. Similarly, at high level of misclassification and endogneity (with a positive $\sigma_{\mu \omega }$ and $\sigma_{\mu \eta }$), the OLS estimator produce estimates that are biased upwards. In the presence of treatment endogeneity without misclassification, as in the case when DNA-fingerprinted adoption data is used (i.e., $\sigma_{\mu \omega }=0.3,\;\sigma_{\mu \eta }=0$ and $\sigma_{\omega \eta }=0$), the OLS estimate is still biased towards zero but the bias is corrected through an IV regression approach. However, in the presence of both endogenious treatment status and misclassification, which corresponds to the use of self-reported adoption data, both the OLS and IV estimates produce biased estimates. These results are consistent with our main empirical findings reported in Section 4.1 and Section 4.2.

**Table 6:** Monte Carlo simulation results.

False negative	False positive	$\sigma_{\mu \omega }$	$\sigma_{\mu \eta }$	$\sigma_{\omega \eta }$	OLS	IV
0	0	0	0	0	0.3	0.3
0	0	0.4	0	0	0.16	0.3
0.47	0.25	0.4	0.1	0.3	0.08	0.2
0.15	0.05	0.01	0.01	0.3	0.1	0.2
0.15	0.05	−0.8	−0.3	−0.3	−0.23	0.19
0.15	0.05	0.8	0.3	0.3	0.54	0.3
0.3	0.05	0.01	0.01	0.3	0.05	0.2
0.3	0.05	−0.8	−0.3	−0.3	−0.36	0.15
0.3	0.05	0.8	0.3	0.3	0.49	0.25
0.3	0.1	0.01	0.01	0.3	0.06	0.24
0.3	0.1	−0.8	−0.3	−0.3	−0.37	0.16
0.3	0.1	0.8	0.3	0.3	0.53	0.24
0.3	0	0.01	0.01	0.3	0.08	0.18
0.3	0	−0.8	−0.3	−0.3	−0.39	0.16
0.3	0	0.8	0.3	0.3	0.57	0.24
0.5	0	0.01	0.01	0.3	0	0.17
0.5	0	−0.8	−0.3	−0.3	−0.52	0.14
0.5	0	0.8	0.3	0.3	0.58	0.19

Results are from 1000 replications with a sample size of 10,000.

$\sigma_{\mu \omega }$ and $\sigma_{\mu \eta }$ denotes the endogeneity of adoption and misclassification, respectively.

$\sigma_{\omega \eta }$ shows the correlation between adoption decision and misclassification.

#### 4.3.3 Plot level estimates

In our final robustness check, we present plot level estimates, focusing on total consumption expenditure. In this case, the treatment variable takes a value of one if the plot is under improved variety and zero otherwise. This is necessary as there is some variation in variety identification within farmers across plots. Reassuringly, results reported in Table 7 are similar with our household level estimates presented in Table 3.

**Table 7:** Plot level estimates: IV results.

	Self-reported		DNA-fingerprinted	
	1	2	3	4
Adoption	0.40^∗∗∗^	0.531^∗∗∗^	0.63^∗∗∗^	0.553^∗∗∗^
	(0.136)	(0.161)	(0.22)	(0.172)
Measurement error ($\eta_{i}$)		-0.363^∗∗∗^		0.18^∗∗^
		(0.08)		(0.095)
Others controls	Yes	Yes	Yes	Yes
Location dummies	Yes	Yes	Yes	Yes
R^2^	0.312	0.366	0.334	0.366
N	5504	5504	5504	5504

Standard errors clustered at the enumeration area-level are reported in parentheses.

Other controls include the variables listed in Table 1.

Significance codes: ‘^∗∗∗^’ 1%,  ‘^∗∗^’ 5%, and ‘^∗^’ 10%.

## 5 Conclusions and implications

In this paper, we revisited the relationship between adoption of improved crop varieties and welfare in a novel way. Traditionally, this relationship has been studied using self-reported adoption data from large household surveys with the assumption that self-reported adoption data reflects the true adoption status of farmers. In this paper, we showed that adoption data from household surveys can be measured with error. Such measurement errors in adoption status can generate spurious findings that have important policy implications. In particular, our empirical results show that measurement errors in self-reported adoption status generate welfare estimates that are biased towards zero and substantially understate the poverty reduction effects of adoption. Furthermore, through simulations, we demonstrated that measurement errors in adoption status can generate upward bias and sign reversal effects. These results have significant implications for drawing robust policy conclusions since policy makers and development practitioners typically use such evidence to make decisions on resource allocations related to technological interventions in the agricultural sector.

In our main analysis, we assumed a non-systematic measurement error in welfare outcome indicators. If these indicators contain systematic errors, then estimates reported in this paper could be biased. While the issue of non-classical measurement error in welfare indicators is beyond the scope of this study, future studies that link innovative data collection approaches for both adoption and welfare indicators would be important to improve the evidence base on the impacts of improved agricultural technologies. Finally, even though the DNA-fingerprinting analysis helps to accurately identify the improvement status of the cassava varieties grown by farmers, classifying varieties into “improved” and “landrace” is not straightforward. In our case, the varieties that were identified as improved through DNA-fingerprinting analysis can be categorized into three broad groups: (i) officially-released improved varieties, (ii) officially released varieties that were not developed through formal breeding processes (i.e., local selections that were released after several years of purification and testing due to their superior characteristics compared to other available local varieties), and (iii) unreleased improved varieties (i.e., farmers often acquire unreleased varieties through backchannels, leakages, and spillovers from research stations). In this regard, examining how differences in key traits and characteristics among the above groups of improved varieties might affect farmers’ adoption decision, variety identification skill, productivity and welfare outcomes would be an important area for future research.

## Appendix A

### A.1 Details on the IV estimator

The relationship expressed in 6 is biased. Note that, by the Law of Iterated Expectation and the relationship presented in Eq. (2), we have the following relationships: $E[T_{i}^{*}|z_{i}]=\mathrm{Pr}[T_{i}^{*}|z_{i}]=\mathrm{Pr}[-\omega_{i}<=z_{i}\varphi ]=F_{\omega }(z_{i}\varphi )$. Let $\theta_{0}=\mathrm{Pr}[\eta_{i}=1|z_{i}]$ and $\theta_{1}=\mathrm{Pr}[\eta_{i}=-1|z_{i}]$ be the probabilities of false positives and negatives respectively. Then $E[T_{i}|z_{i}]=\theta_{0}+[1-\theta_{0}-\theta_{1}]F_{\omega }(z_{i}\varphi )$. From this, it follows that,

(11) δ^iv=δcov(θ0+[1-θ0-θ1]Fω(ziφ),Fω(ziφ))varθ0+[1-θ0-θ1]Fω(ziφ)=δλ≠δ

where,

(12) $$\lambda =\frac{\mathrm{cov}(\theta_{0}+[1-\theta_{0}-\theta_{1}]F_{\omega }(z_{i}\varphi ),F_{\omega }(z_{i}\varphi ))}{\mathrm{var}\left(\theta_{0}+[1-\theta_{0}-\theta_{1}]F_{\omega }(z_{i}\varphi )\right)}\text{.}$$

That is, δ^IV is inconsistent (unless there is no misclassification, (i.e. $\theta_{0}=\theta_{1}=0$)). Moreover, we cannot sign the bias in general, given that we could have λ>1 or λ<1 when $\theta_{0}$ and $\theta_{1}$ are not constant. For the true adoption data (i.e. from DNA-fingerprinting), we have ($\theta_{0}=\theta_{1}=0)$ and hence the IV estimator in Eq. (11) is unbiased (i.e. δ^iv∗=δ).

### A.2 Details on measurement error in the dependent variable

Suppose further, that the measurement error $\Psi_{i}$ is non-classical such that we can write $\Psi_{i}=\alpha W_{i}^{*}+\nu_{i}$. Then the relationship that we estimate using observed consumption expenditure becomes:

(13) $$W_{i}=(1+\alpha )W_{i}^{*}+\nu_{i}$$

which can be re-written as

(14) $$W_{i}=\delta (1+\alpha )T_{i}^{*}+(1+\alpha )\mu_{i}+\nu_{i}$$

In the presence of non-classical measurement error in Wi∗,δ^ols becomes:

(15) δ^ols=cov(Wi,Ti∗)var(Ti∗)=δ(1+α)σT∗2+(1+α)σT∗μ+σT∗νσT∗2=δ(1+α)+(1+α)σT∗μ+σT∗νσT∗2

Note that, if both consumption expenditure and adoption status suffer from non-classical measurement error and if such errors are correlated the bias in δ^ols is given by:

(16) δ^ols=cov(Wi,Ti)var(Ti)=cov(δ(1+α)Ti∗+(1+α)μi+νi,Ti∗+ηi)var(Ti∗+ηi)=δ(1+α)σT∗2+δ(1+α)σT∗η+(1+α)σT∗μ+(1+α)σημ+σην+σT∗νσT∗2+2σT∗η+ση2.

## Footnotes

1 Improved production allows adopters to consume some of their own produce.

2 We assume that measurement error reflects misreporting instead of misperception.

3 In fact, since $E[W_{i}]=(1+\delta )E[W_{i}^{*}]$, the sign of the bias depends on how the average reported consumption compares to the average true consumption. If the former is lower than the latter, there is downward bias, and there is upward bias otherwise. However, just like income, true low-consumers may tend to over-report consumption whereas true high-consumers may tend to under-report consumption (mean-reverting measurement errors).

4 Details on the bias associated with other possible measurement errors, including correlated non-classical measurement error, are described in the appendix.

5 Information about the process of the DNA-fingerprinting process is presented in online supplementary material.

6 The intensification rate, total cassava area under improved varieties is about 39%.

7 Note that, these are not the conditional probabilities. For example the conditional probability of false negatives is about 30%.

8 Household level correlates of correct identification of improved varieties is reported in supplementary material. We show that farmers with access to formal sources of planting material, access to more structured sources of information (mobile phone ownership, access to extension access, and formal and informal associations), and with a better educational level are more likely to identify the improved varieties they grow. Details are presented in (Wossen et al., 2019).

9 Details about alternative improved variety classification approaches are presented in Wossen et al. (2017) and can be accessed here: https://cgspace.cgiar.org/handle/10568/80706.

10 These indicators can be prone to measurement error. For example, comparing recall based consumption data collection approaches with the benchmark personal diary approach, Beegle et al. (2012), documented significant differences between the two approaches.

11 The Food and Agriculture Organization of the United Nations, identify the four pillars of food security as availability, access, utilization, and stability.

12 Regression results with the full list of controls are presented in supplementary material.

13 If one has a relevant and exogenous instrument for measurement error, then IV estimates will be unbiased.

14 First stage results are presented in supplementary material.

15 Coefficients for food shortage are treatment effects from bivariate probit model.

16 We used a biprobit model since both the dependent and the treatment (adoption) variables are binary.
