# Supplementary material for: Agricultural technology adoption and household welfare: Measurement and evidence
Source: Food Policy. 2019 Aug;87:101742. doi: 10.1016/j.foodpol.2019.101742 (PMC6988438; doi:10.1016/j.foodpol.2019.101742)
Supplement: Supplementary data 1 [file mmc1.zip › ecomponent/DNA_fingerprinting.docx]

**Summary of the DNA-fingerprinting process**

Several options exist in varietal identification for tracking the adoption of improved varieties. However most of these methods have inherent uncertainty levels and estimates often have wide confidence intervals. This paper therefore uses DNA-fingerprinting based varietal identification. For this study, DNA was isolated following the DNA extraction protocol (Dellaporta *et al*., 1983) from a total of 7376 genotypes collected from 2500 household’s including 89 samples for quality control (clones genotyped in duplicate). In house modified protocol (Rabbi *et al*., 2014) that enables to extract up to 10 plates of 96 samples each per day was implemented. All the extracted DNA samples were quantified using spectrophotometer and agarose gel electrophoresis for quality and quantity assessments. Furthermore, test digestion with restriction enzyme was performed for 10% of the samples extracted as suggested by Genomic Diversity Facility (GDF) at Cornell University for standard Genotyping by Sequencing (GBS) library preparation. DNA samples with high concentration were diluted to 1000ng/μl. All extracted samples that pass the minimum quantity requirement (300ng/μl) were shipped to GDF for genotyping by sequencing (GBS). The ApeKI restriction enzyme (recognition site: G|CWCG) that produces less variable distributions of read depth was used for the GBS library preparation and therefore a larger number of scorable SNPs in cassava were used. Eighty 96-plex GBS libraries were constructed following the standard procedure (Elshire et al. 2011) and sequenced at the GDF using the Illumina HiSeq2500.

The raw read sequences obtained in the current study including accessions in the reference library (Rabbi et al 2015) and duplicate of 89 samples for quality control were processed through a TASSEL-GBS discovery pipeline developed using TASSEL 5.0 (Glaubitz et al. 2014). SNP calling was performed based on TASSEL-GBS production pipeline by aligning the tags to the most recent cassava reference genome version 6.0. The 89 randomly selected and genotyped in duplicates were used to determine a distance threshold between genotypes that can help to declare a distance at which two or a set of genotypes are similar or distinct. A frequency distribution of distance (IBS) was plotted and resulted in bimodal distribution of pairwise genetic distance. The bimodal distribution shows the frequency distribution of the data where one of the curves shows artefact that could occur due to genotyping error. The point between the bimodal distributions was therefore declared as a distance threshold where any pair of genotypes or set of genotypes below the point are identical.

Once the distance threshold is determined, the distance-based hierarchical clustering, a pairwise genetic distance (identity-by-state, IBS) matrix were computed for all the genotypes including the intentional duplicates and accessions from the reference library (Rabbi et al, 2005). A Ward’s minimum variance hierarchical cluster dendrogram were built from the IBS matrix. The critical distance threshold determined was applied for the whole data and individuals belonging to the same cluster group below the threshold were considered as the same genotypes, i.e. if any of the genotypes from the reference library fall in the cluster of different individuals representing the same variety then it will be identified based on the variety from the reference library. Details about the DNA-fingerprinting process are presented below.

**Overview of the DNA fingerprinting workflow**

The DNA fingerprinting component of the CMS involved establishment of a clear workflow which could be used as a reference for similar studies addressing the tracking of adoption of improved varieties. The workflow was as follows:

1. Establishment of a reference library comprising improved varieties, the genebank collection, and landraces.
2. Field sample collection and preservation.
3. Establishment of tracking system to ensure the chain of custody of sample identification from field to laboratory.
4. High throughput DNA extraction that allows extraction daily from a large number of samples.
5. Genotyping.
6. Bioinformatics and cultivar identification.

**Sample and sample associated data collection**

Leaf samples for all the varieties identified by farmers in each household were collected and preserved in plastic tubes containing silica gel and transported to the Bioscience laboratory at IITA in Ibadan, for DNA extraction. Household information including ID for region, state, local government area, EA, and household as well as the name of the household head was captured in a booklet. In addition, information on variety name, cropping pattern (mono-cropped or intercropped), field and plot identification, plot size in all the fields owned by the household was recorded and the GPS coordinates were measured of the household where the survey took place and the farmers’ fields.

A standard tracking system is important particularly when dealing with a large sample size to reduce any possible introduction of human errors of sample mismatch and mix ups. A tracking system with multiple layers was implemented using barcode labels, self-adhesive stickers, booklet and tablet computers for capturing samples and sample-associated information. This process has improved the accuracy and reliability of the data. Duplicate barcodes were prepared and pasted both on sample collection tube and booklet for each sample collected. Once received in the lab samples were arranged in a set of 96 on a plate made in-house and assigned distinct plate numbers. Both plate and sample numbers were written on the cover of each sample collection tube and were different for the 96 samples. Information on each vial consisting of the barcode label and other metadata was captured manually on a tablet and on a hard copy plate map. The barcode label on each tube was also captured with a barcode reader in parallel using DNA plate software obtained from [www.wheatgenomics.org](http://www.wheatgenomics.org). Manually entered and barcode reader information was cross-checked. This has helped a lot in reducing the possible introduction of human errors or sample mix ups.

Field samples included leaf tissue collection, preservation in plastic tubes containing silica gel/desiccant and the recording of sample-associated information. Due consideration and intensive training were given to enumerators prior to the field visit and on the field to ensure proper sampling, conservation of plant tissue, and capture of sample-related information. Before field visits, more than 8000 plastic tubes of 50 ml size containing 20 g silica gel were prepared and adhesive labels with adequate space to capture information were pasted on all tubes. In addition, a barcode label unique for each variety was prepared in duplicate and pasted on the sample collection tubes and booklets. Other sample-associated information was also captured. A total of 7428 different samples were collected from 2500 households of the four regions of Nigeria

**DNA extraction and genotyping by sequencing**

DNA was isolated following the extraction protocol of Dellaporta et al. (1983) with some modification for the large-scale sample from a total of 7428 genotypes collected from 2500 households including quality control (89 clones genotyped in duplicate) and re-extracted samples. In-house modified protocol (Rabbi et al. 2014) was implemented that enables up to 10 plates of 96 samples each to be extracted per day. All the extracted DNA samples were quantified using the spectrophotometer and agarose gel electrophoresis for quality and quantity assessments. Test digestion with restriction enzyme was performed for 10% of the samples extracted as suggested by the Genetic Diversity Facility (GDF) at Cornell University for standard Genotyping by Sequencing (GBS) library preparation. DNA samples with high concentrations were diluted to 1000 ng/μl. All extracted samples that passed the minimum quantity requirement (300 ng/μl) were shipped to GDF for GBS. Samples were originally processed in three batches consisting of 30 plates for the first two batches and 20 plates for the third. A few of the total sequenced samples from Batch had a low pass count (< 500,000 reads). These samples were therefore re-extracted and included in the third batch. As a result, a total of 7565 samples were genotyped including 89 samples genotyped in duplicate. The purpose of genotyping samples in duplicate was to identify any possible SNP genotype error resulting from miscalling some heterozygous SNPs with low sequencing read depth as homozygotes and to determine a threshold to declare if two genotypes are the same or different.

For GBS library preparation, the ApekI restriction enzyme (recognition site: G|CWCG) was used that produces less variable distributions of read depth and therefore a larger number of scorable SNPs in cassava. Eighty 96-plex GBS libraries were constructed following the standard procedure (Elshire et al. 2011) and sequenced at the Institute of Genomic Diversity at Cornell University using the Illumina HiSeq2500.

### **Development of a reference library for varietal identification**

Developing a well-curated comprehensive reference library in collaboration with the breeding programs is very important for tracking genotypes of interest, otherwise DNA fingerprinting alone can be used only to establish baseline data. The quality of the reference library (genotype traceability, and comprehensiveness) determines the “level of success” in varietal identification. The already available reference library with a total of 3891 diverse genotypes comprising a collection of known improved lines, IITA regional breeding program, IITA germplasm collection, wild species, and CIAT collection were used in the current study. The GBS technique was applied for the development of the reference library. Likewise, the GBS procedure was implemented for the genotyping of the current samples collected from farmers for varietal identification. Raw sequences of the two data, consisting of accessions in the reference library and the current samples collected from farmers’ fields, were combined for single nucleotide polymorphism (SNP) calling and further identification and analysis of varieties.

**Bioinformatics**

An initial raw sequence data of approximately 200 Gbp were generated after sequencing 7565 genotypes. The raw read sequences of the samples in the current study were processed in combination with the accessions in the reference library through cassava TASSEL-GBS discovery pipeline (Fig. below) developed using TASSEL 5.0 and initially generated with about 2500 cassava clones under the NextGen Cassava project ([www.nextgencassava.org](http://www.nextgencassava.org)). A SNP calling was performed based on TASSEL-GBS production pipeline by aligning the tags to the most recent cassava reference genome, version 6.0.


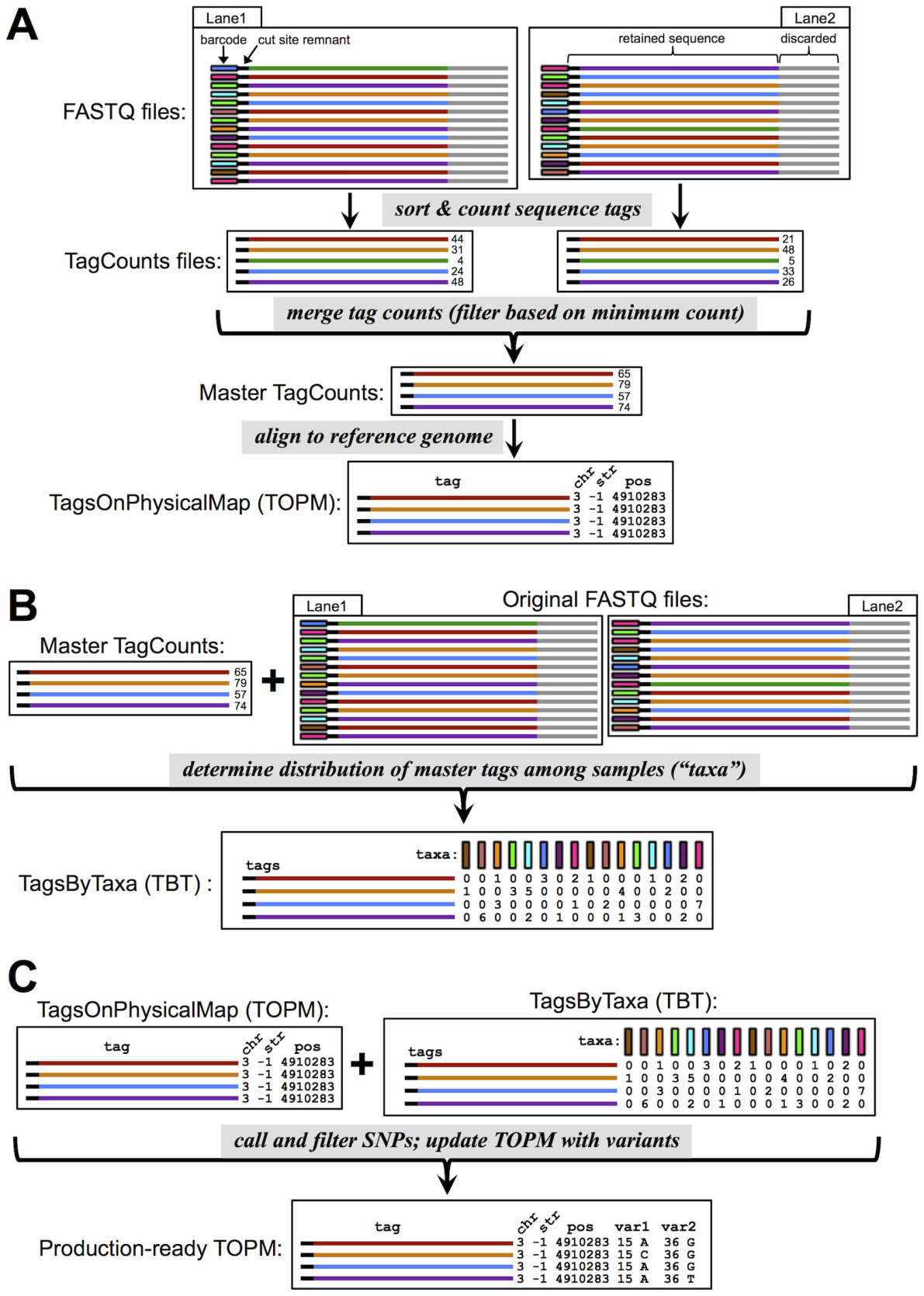


Schematic representation of the TASSEL-GBS Discovery Pipeline adapted from Glaubitz et al. 2014.

*(A) Barcoded sequence reads are processed and collapsed into a set of unique sequence tags, with one TagCounts file produced per input FASTQ file. The separate TagCounts files are then merged to form a ‘‘master’’ TagCounts file, which retains only those tags present at or above an experiment-wide minimum count. This master tag list is then aligned to the reference genome and a TagsOnPhysicalMap (TOPM) file is generated, containing the genomic position of each tag with a unique, best alignment. (B) The barcode information in the original FASTQ files is then used to tally the number of times each tag in the master tag list is observed in each sample (‘‘taxon’’) and these counts are stored in a TagsByTaxa (TBT) file. (C) The information recorded in the TOPM and TBT is then used to discover SNPs at each ‘‘TagLocus’’ (set of tags with the same genomic position) and filter the SNPs based upon the proportion of taxa covered by the TagLocus, minor allele frequency, and inbreeding coefficient (FIT). For each retained SNP, the allele represented by each tag in the corresponding TagLocus is recorded in the TOPM file, along with its relative position in the locus. The end product of the Discovery Pipeline is a ‘‘production-ready’’ TOPM that can then be used by the Production Pipeline to call SNPs.*


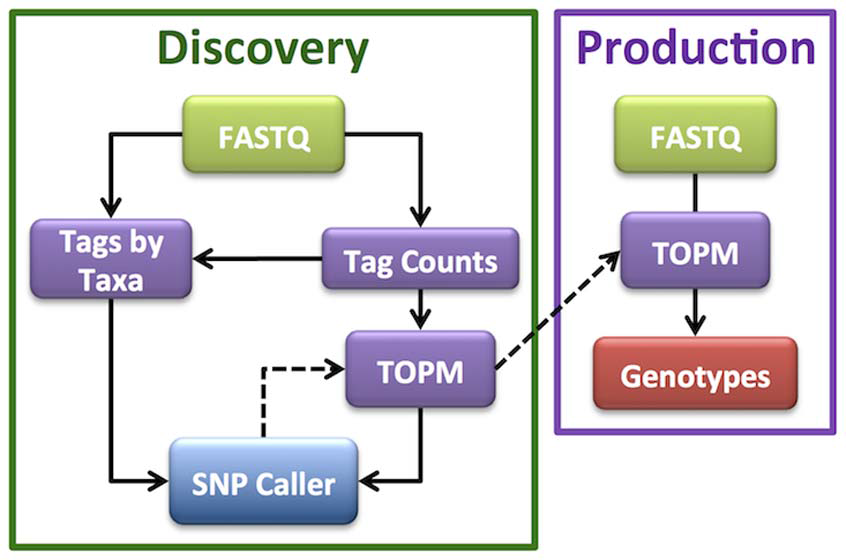


The TASSEL-GBS production pipeline and its relationship with discovery pipeline. Adapted from Glaubitz et al. 2014

*The Discovery Pipeline is run periodically on all FASTQ files generated to date in a species, and the ascertained and filtered SNPs are stored in a ‘‘production-ready’’ TOPM. The Production pipeline utilizes this production-ready TOPM to quickly call SNPs either for the original samples in the Discovery Build, or for subsequent, post-Discovery samples.*

Resulting hapmap files (SNPs) were filtered for quality control of SNP data by removing those with > 30% missing data and minor allele frequency of < 0.01%. A total of 62 548 single nucleotide variants (SNV) were initially discovered from 11 578 accessions consisting of 3891 of the reference library samples and 7411 of the CMS samples. Filtering as a quality control measure was done by missing genotype rates at maximum per-variant == 0.6 and maximum per-sample == 0.8. A total of 276 accessions and 9649 SNV were removed owing to missing data. The final data therefore consisted of 52 899 variants and 11 302 accessions that passed filters and quality control.

### **Distance threshold to establish criteria to determine identical sets of genotypes**

A total of 89 samples randomly selected were genotyped in duplicate to determine a distance threshold between genotypes that could help to declare a distance at which two or a set of genotypes are similar or distinct. A frequency distribution of distance (IBS) was plotted and resulted in bimodal distribution of pairwise genetic distance. The bimodal distribution shows the frequency distribution of the data where one of the curves shows an artefact that could occur owing to genotyping error. Filtering with minor allele frequency (MAF) at 0.00025, missing individuals at 0.8, and missing genotypes of 0.6 was done and resulted in a total genotyping rate of 0.642083 with total variants of 35729. A frequency histogram of distance then resulted in a distance threshold value of 0.0166. The red dashed line at 0.0166 as indicated on the dendrogram was therefore declared as a distance threshold where any pair of genotypes or set of genotypes below the line or less than 0.0166 are identical (Fig below).

Established criteria for determining identical sets of clones based on 89 samples genotyped in duplicates.

**Cluster analysis**

Identification of cassava varieties collected from farmers’ fields was first performed using distance-based hierarchical clustering, a pairwise genetic distance (identity-by-state, IBS) matrix calculated from 52,899 SNP markers in PLINK: Whole genome data analysis toolset version 1.9 (Purcell et al., 2007). A Ward’s minimum variance hierarchical cluster dendrogram was then built from the IBS matrix using the Analyses of Phylogenetics and Evolution (ape) package in R software. The critical distance threshold to declare whether two genotypes are identical was empirically determined from the distribution of pairwise distances between duplicated DNAs from 89 samples. This “calibration principle” approach was taken because of the possibility of SNP genotype errors resulting from miscalling as homozygotes some heterozygous SNPs with low sequencing read depth.

### **Matching farmers’ varieties to those in the reference library**

The hierarchical clustering of 11302 accessions based on Ward’s minimum variance revealed two main cluster groups representing the genetic gain collection and IITA germplasm collections. The genetic gain and germplasm collections have also prefix names: TMS representing breeding lines and TME representing the gene bank collection. Individual accessions in the same cluster with the distance threshold below 0.0166 were all considered as the same genotypes. Any genotype from the reference library that falls in the cluster of different individuals representing the same variety is identified based on the reference library. Of the total 7376 farmers’ varieties collected in the current study only 4822 matched genotypes in the reference library, whereas 2554 did not match any of the varieties there. On the other hand, 1663 of the 3891 genotypes in the reference library did not match any of the varieties in CMS. Those varieties not matching the genotypes in the reference library were further observed for any match to the genetic gain cluster groups so that their improvement status could be considered. Altogether a total of 114 different varieties were identified (Table below). Among these, 46 varieties matched the genetic gain cluster group whereas 68 matched landrace groups, 18 matched officially released varieties, and 15 matched varieties that are improved and released. Among the officially released varieties only 14 were from the genetic gain group whereas the remaining four cultivars represented landrace collections evaluated on experimental plots and officially released.

Table. List of genotypes in the reference library matching farmers’ field collected varieties with its respective genetic group, release information, and improvement status.

| Matched cultivar | Classification (GG or LR) | Matched released varieties | Status [improved/improved + released/released] |
| --- | --- | --- | --- |
| TMEB1116/TMEB99/TMEB1135/TMEB1105/TMEB1099/TMEB1212 | GG | NO | Improved |
| TMSI920326 | LR | TMS 92/0326 | Improved + Released |
| TMEB597 | LR | NO | NO |
| AKPU/TMEB1142 | LR | NO | NO |
| TMEB2/ODONGBO | LR | MS-3 (Odongbo) | Released |
| TMS30572 | GG | TMS-30572 | Improved + Released |
| IITA_GRU_LR_core_258 | GG | NO | LR + Match GG |
| TMEB966/TMEB965 | LR | NO | NO |
| TMSI980581 | GG | TMS 98/0581 | Improved + Released |
| TMEB419 | LR | TME-419 | Released |
| TMEB1044/TMEB1042 | LR | NO | NO |
| TMEB1641/TMEB265/TMEB1643/TMEB1992/TMEB1774 | LR | NO | NO |
| TMSI9102324 | GG | NO | Improved |
| TMSI010760 | LR | NO | Improved |
| TMEB104/TMEB102/TMEB37 | LR | NO | NO |
| TMEB971/TMEB1736 | LR | NO | NO |
| I9102325 | GG | NO | Improved |
| TMEB3, 4,7,12,14 | LR | NO | NO |
| ANTIOTA/TMEB1 | LR | MS-6 (Antiota) | Released |
| TMEB1369/TMEB634/TMEB813 | LR | NO | NO |
| CW5251/NR8082 | GG | NR-8082 | Improved + Released |
| BEN 86019/Caricass/Toma | LR | NO | NO |
| TMEB1040/TMEB1050 | LR | NO | NO |
| TMEB117/ANKRA | LR | NO | NO |
| TMSI980505 | GG | TMS 98/0505 | Improved + Released |
| TMSI071313 | GG | NO | Improved |
| MBOMA11 | LR | NO | NO |
| TMEB491/TMEB508 | LR | NO | NO |
| TMEB1858/TMEB119/TMEB1024 | LR | NO | NO |
| TMSI011368 | GG | IITA TMS 1011368 | Improved + Released |
| TMSI4(2)1425 | GG | TMS-4(2)1425 | Improved + Released |
| GRU_LR_core_157 | LR | NO | NO |
| TMEB262/TMEB1647/TMEB410/TMEB2048 | LR | NO | NO |
| TMEB1000 | LR | NO | NO |
| TMSI940270 | LR | NO | Improved |
| NR7734 | GG | NO | Improved |
| TMEB120 | LR | NO | NO |
| Malawi_034 | GG | NO | LR + Match GG |
| TMEB33 | LR | NO | NO |
| TMEB621 | LR | NO | NO |
| TMEB47 | LR | NO | NO |
| TMSI30040 | LR | NO | Improved |
| TMEB8 | LR | NO | NO |
| TMEB34 | LR | NO | NO |
| TMEB131 (C-2 Cape Verde) /TMEB2050/TMEB994 | LR | NO | NO |
| TMSI011412 | GG | IITA TMS 1011412 | Improved+ Released |
| AR1-81/CR12-45/AR37-108 (TME961-WarriNorthDelta) | LR | NO | Improved |
| GRU_LR_core_198 | LR | NO | NO |
| TMEB470/TMEB625/TMEB2089/TMEB2056/TMEB933 | LR | NO | NO |
| Cameroon_010 | LR | NO | NO |
| Cameroon_030 | LR | NO | NO |
| TMEB10 | LR | NO | NO |
| MOCUBA | LR | NO | NO |
| TMS50395 | GG | TMS-50395 | Improved + Released |
| TMEB1818 | LR | NO | NO |
| TMEB463/235 | LR | NO | NO |
| TMEB1200/TMEB983 | GG | NO | LR + Match GG |
| TMEB1485/TMEB1278 | LR | NO | NO |
| TMSI920057 | GG | TMS 92/0057 | Improved + Released |
| W940727 | GG | NO | Improved |
| TMEB2122 | LR | NO | NO |
| TMEB945 | LR | NO | NO |
| TMSI000354 | GG | NO | Improved |
| TMEB9 | GG | NO | LR + Match GG |
| Ug130005 | GG | NO | LR + Match GG |
| Ug120078 | LR | NO | NO |
| TMSI011752 | LR | NO | Improved |
| TMSI30555/NR8212 | GG | TMS-30555 | Improved + Released |
| TMSI960325 | GG | NO | Improved |
| TMSZ010087 | LR | NO | Improved |
| TMEB981 | LR | NO | NO |
| TMSI982101 | GG | NO | Improved |
| Ug120140 | GG | NO | LR + Match GG |
| Ug120142 | GG | NO | LR + Match GG |
| TMSI972205 | GG | TMS 97/2205 | Improved + Released |
| B9200068 | LR | NO | Improved |
| TMSI011371 | GG | IITA TMS 1011371 | Improved + Released |
| SLICASS3 | LR | NO | NO |
| TMEB469/TMEB468/TMEB2023/TMEB2021/TMEB2068 | LR | NO | NO |
| TMSI30211/I40764 | GG | NO | Improved |
| TMEB148 | GG | NO | LR + Match GG |
| TMSI980002 | GG | TMS 98/0002 | Improved + Released |
| TMEB286 | LR | NO | NO |
| TMEB1386/TMEB1484/TMEB1509 | LR | NO | NO |
| TMEB1321 | GG | NO | LR + Match GG |
| CR14B218 | GG | NO | Improved |
| TMSI020452 | GG | NO | Improved |
| TMEB499 | LR | NO | NO |
| TMEB23/TMEB24 | LR | NO | NO |
| TMEB279/TMEB624 | LR | NO | NO |
| TMSI60506 | GG | NO | Improved |
| TMEB121 | LR | NO | NO |
| TMEB1019/TMEB1002/TMEB1046/TMEB1021/TMEB1006 | LR | NO | NO |
| TMEB30/TMEB31/TMEB21 | LR | NO | NO |
| AR9-19 | LR | NO | Improved |
| TMSI960304/TMSI960804 | LR | NO | Improved |
| TMEB1001 | LR | NO | NO |
| COB1139 | GG | NO | Improved |
| TMEB2121 | GG | NO | LR + Match GG |
| TMSI9102322 | LR | NO | Improved |
| TMSI974779 | GG | NO | Improved |
| TMEB497/TMEB1025 | LR | NO | NO |
| I083389/I940561 | GG | NO | Improved |
| B9200061 | LR | NO | Improved |
| NR050080 | GG | NO | Improved |
| CW45113 | LR | NO | Improved |
| TMSI011646 | GG | NO | Improved |
| NR090146/NR110122/NR87184 | GG | NR 87184 | Improved + Released |
| TMSI011811/I010536 | GG | NO | Improved |
| NZIVA | GG | NO | LR + Match GG |
| TMEB762/TMEB757 | LR | NO | NO |
| TMEB960 | LR | NO | NO |
| TMEB952 | GG | NO | LR + Match GG |
| Ug120145 | GG | NO | LR + Match GG |

GG = genetic gain; LR = land race; LR + Match GG = varieties matching landrace in the genetic gain group.
