# Supplementary material for: Agricultural technology adoption and household welfare: Measurement and evidence
Source: Food Policy. 2019 Aug;87:101742. doi: 10.1016/j.foodpol.2019.101742 (PMC6988438; doi:10.1016/j.foodpol.2019.101742)
Supplement: Supplementary data 1 [file mmc1.zip › ecomponent/Supplemntary Material_Full_Tables.pdf]

Online supplementary material for: Agricultural Technology Adoption and Household Welfare: Measurement and Evidence

Tesfamicheal Wossen[†*], Arega Alene[†], Tahirou Abdoulaye[†], Shiferaw Feleke[†], and Victor Manyong[†]

[†]International Institute of Tropical Agriculture (IITA)

[*]Corresponding author: Tesfamicheal Wossen: t.assfaw@cgiar.org

This supplementary material contains the following items:

1) First stage regression results
2) The full regression results of the OLS regression (Table 2)
3) The full regression results of the IV regression (Table 3)

Table 1: First stage regression results

	Self-reported adoption	DNA-adoption
Household size	0.038**	0.029*
	(0.016)	(0.015)
Number of boys below 12	0.065**	0.023
	(0.027)	(0.024)
Number of girls below 12	0.005	-0.021
	(0.025)	(0.022)
Education	0.001	0.014**
	(0.007)	(0.006)
Age	0.002	0.006**
	(0.003)	(0.003)
Sex	0.027	-0.271***
	(0.107)	(0.103)
TLU	0.001	0.015**
	(0.002)	(0.006)
Value of asset	-0.031*	-0.000
	(0.017)	(0.014)
Ownership of television	0.005	-0.021
	(0.092)	(0.076)
Ownership of mobile phoes	0.421***	0.336**
	(0.159)	(0.158)
Access to off-farm	0.432***	0.093
	(0.077)	(0.071)
Access to extension	-0.032	-0.012
	(0.079)	(0.068)
Access to credit	0.340***	0.086
	(0.093)	(0.082)
Membership in cassava association	0.165**	0.003
	(0.076)	(0.068)
Membership in credit and saving organization	0.188**	-0.021
	(0.083)	(0.079)
Membership in cooperatives	-0.000	-0.002
	(0.000)	(0.002)
Total farm size	0.036	0.054
	(0.028)	(0.033)
Quality of road	-0.003	-0.002
	(0.005)	(0.006)
Distance from village market	-0.002	-0.001
	(0.002)	(0.003)
Distance from district market	-0.002	-0.002
	(0.003)	(0.003)
Incidence of cassava pests	1.267***	0.373***
	(0.073)	(0.065)
Fufu and Gari preference	0.874***	0.826***
	(0.218)	(0.273)
Access to stem markets	0.726***	0.573***
	(0.111)	(0.109)
Observations	2,214	2214
Pseudo R2	0.36	0.17

State dummies included but not reported here due to space limitation.

Table 2: Full OLS estimates

	Self-reported			DNA-fingerprinted		
	Food shortage	Food Exp.	Total Exp.	Food shortage	Food Exp.	Total Exp.
Adoption	-0.262***	0.191***	0.142***	-0.34***	0.353***	0.284***
	(0.025)	(0.041)	(0.035)	(0.022)	(0.034)	(0.029)
Household size	-0.030**	-0.172***	-0.158***	-0.030**	-0.173***	-0.159***
	(0.014)	(0.009)	(0.007)	-0.014	(0.009)	(0.007)
Number of boys below 12	0.030	0.035***	0.030***	0.027	0.035***	0.030***
	(0.024)	(0.013)	(0.011)	-0.024	(0.012)	(0.010)
Number of girls below 12	0.015	0.016	0.010	0.008	0.019	0.012
	(0.023)	(0.013)	(0.011)	-0.023	(0.013)	(0.011)
Education	-0.018***	0.005	0.008***	-0.014**	0.004	0.007**
	(0.006)	(0.004)	(0.003)	-0.006	(0.004)	(0.003)
Age	-0.007***	-0.002	-0.002**	-0.006**	-0.002*	-0.003**
	(0.002)	(0.001)	(0.001)	-0.002	(0.001)	(0.001)
Sex	-0.057	0.049	0.057	-0.164*	0.084	0.085*
	(0.100)	(0.055)	(0.045)	-0.099	(0.054)	(0.044)
TLU	-0.014**	-0.000	0.000	-0.011**	-0.000	0.000
	(0.005)	(0.001)	(0.001)	-0.005	(0.001)	(0.001)
Value of asset	-0.032**	0.019**	0.028***	-0.027*	0.018**	0.027***
	(0.015)	(0.008)	(0.007)	-0.015	(0.008)	(0.007)
Ownership of television	-0.045	0.096**	0.093**	-0.064	0.098**	0.094***
	(0.075)	(0.043)	(0.037)	-0.075	(0.042)	(0.036)
Ownership of mobile phones	-0.138	-0.032	0.054	-0.172	-0.043	0.043
	(0.170)	(0.095)	(0.077)	-0.158	(0.093)	(0.076)
Access to off-farm	-0.432**	0.389***	0.299***	-0.292	0.296***	0.221***
	(0.194)	(0.099)	(0.086)	-0.186	(0.097)	(0.085)
Access to extension	-0.008	0.062*	0.036	-0.06	0.071**	0.042
	(0.065)	(0.037)	(0.030)	-0.065	(0.036)	(0.030)
Access to credit	-0.143**	0.036	0.038	-0.148**	0.036	0.038
	(0.067)	(0.038)	(0.032)	-0.069	(0.037)	(0.031)
Membership in cassava association	-0.023	0.078**	0.074**	-0.064	0.083**	0.077**
	(0.075)	(0.040)	(0.033)	-0.076	(0.039)	(0.032)
Membership in cre-saving organization	0.045	-0.000	0.020	0.016	0.007	0.026
	(0.065)	(0.037)	(0.031)	-0.068	(0.037)	(0.031)
Membership in cooperatives	-0.132	0.401***	0.362***	-0.174	0.361***	0.326***
	(0.185)	(0.104)	(0.085)	-0.183	(0.107)	(0.085)
Total farm size	-0.011	0.204**	0.246***	-0.269	0.240***	0.271***
	(0.177)	(0.088)	(0.074)	-0.183	(0.082)	(0.070)
Quality of road	-0.227	0.308***	0.288***	0.063	0.175*	0.179**
	(0.188)	(0.090)	(0.080)	-0.184	(0.096)	(0.085)
Distance from village market	0.008	0.002	0.003	0.007	0.002	0.003
	(0.006)	(0.003)	(0.003)	-0.005	(0.003)	(0.003)
Distance from district market	-0.002	0.000	-0.000	-0.002	0.000	0.000
	(0.002)	(0.001)	(0.001)	-0.002	(0.001)	(0.001)
Observations	2,214	2214	2,214	2214	2,214	2,214

State dummies included but not reported here due to space limitation.

Table 3: Full IV estimates

	Self-reported			DNA-fingerprinted		
	Food shortage	Food Exp.	Total Exp.	Food shortage	Food Exp.	Total Exp.
Adoption	-0.238***	0.410***	0.287**	-0.364***	0.592**	0.414**
	(0.086)	(0.149)	(0.122)	(0.105)	(0.265)	-0.179
Household size	-0.031**	-0.174***	-0.160***	-0.029*	-0.176***	-0.161***
	(0.016)	(0.009)	(0.007)	(0.015)	(0.009)	(0.007)
Number of boys below 12	0.028	0.031**	0.027**	0.027	0.033***	0.028***
	(0.023)	(0.013)	(0.011)	(0.023)	(0.012)	(0.010)
Number of girls below 12	0.015	0.016	0.010	0.007	0.020	0.013
	(0.022)	(0.012)	(0.011)	(0.024)	(0.013)	(0.012)
Education	-0.018***	0.005	0.008***	-0.014*	0.002	0.006*
	(0.007)	(0.004)	(0.003)	(0.007)	(0.004)	(0.003)
Age	-0.007***	-0.002	-0.003**	-0.006**	-0.003*	-0.003***
	(0.002)	(0.001)	(0.001)	(0.003)	(0.001)	(0.001)
Sex	-0.058	0.046	0.055	-0.172	0.106*	0.100**
	(0.099)	(0.055)	(0.045)	(0.106)	(0.059)	(0.048)
TLU	-0.013	-0.000	0.000	-0.011	-0.001	-0.000
	(0.021)	(0.001)	(0.001)	(0.022)	(0.001)	(0.001)
Value of asset	-0.031**	0.021**	0.029***	-0.027*	0.018**	0.027***
	(0.016)	(0.008)	(0.007)	(0.015)	(0.008)	(0.007)
Ownership of television	-0.046	0.095**	0.092**	-0.064	0.099**	0.095***
	(0.079)	(0.043)	(0.037)	(0.077)	(0.042)	(0.036)
Ownership of mobile phones	-0.149	-0.061	0.035	-0.165	-0.068	0.026
	(0.164)	(0.097)	(0.078)	(0.170)	(0.098)	(0.078)
Access to off-farm	-0.311	0.376***	0.311**	-0.198**	0.325***	0.290**
	(0.123)	(0.097)	(0.123)	(0.099)	(0.118)	(0.146)
Access to extension	-0.018	0.038	0.019	-0.059	0.064*	0.036
	(0.070)	(0.041)	(0.034)	(0.069)	(0.037)	(0.031)
Access to credit	-0.142**	0.037	0.039	-0.147**	0.036	0.039
	(0.067)	(0.038)	(0.031)	(0.068)	(0.037)	(0.031)
Membership in cassava association	-0.030	0.060	0.062*	-0.061	0.076*	0.072**
	(0.076)	(0.041)	(0.034)	(0.078)	(0.039)	(0.032)
Membership in cre-saving organization	0.042	-0.010	0.014	0.017	0.006	0.025
	(0.069)	(0.037)	(0.032)	(0.071)	(0.037)	(0.032)
Membership in cooperatives	0.042	0.018	0.010	0.001	0.040	0.025
	(0.072)	(0.038)	(0.032)	(0.077)	(0.037)	(0.031)
Total farm size	0.031	0.931***	0.826***	0.000	0.723***	0.874***
	-0.01	(0.243)	(0.273)	(0.002)	(0.192)	(0.218)
Quality of road	-0.011	0.023	0.017	0.001	0.017	0.012
	(0.025)	(0.016)	(0.013)	(0.027)	(0.017)	(0.014)
Distance from village market	0.008	0.002	0.003	0.007	0.002	0.003
	(0.006)	(0.003)	(0.003)	(0.006)	(0.003)	(0.003)
Distance from district market	-0.001	0.000	0.000	-0.001	-0.000	0.000
	(0.002)	(0.001)	(0.001)	(0.002)	(0.001)	(0.001)
Observations	2,214	2214	2,214	2214	2,214	2,214

State dummies included but not reported here due to space limitation.
